# PROGNOSTIC MARKERS FOR THROMBOTIC EVENTS IN PATIENTS WITH GASTRIC OR COLORECTAL ADENOCARCINOMAS

**DOI:** 10.1590/0102-6720202400039e1833

**Published:** 2024-11-25

**Authors:** Emilly de Assis MACHADO, Marcelo Gerardin Poirot LAND, Alberto SCHANAIDER

**Affiliations:** 1 Universidade Federal do Rio de Janeiro, Department of Surgery – Rio de Janeiro (RJ), Brazil.

**Keywords:** DNA, Thrombin, Prothrombin, Thrombosis, Colorectal neoplasms, DNA, Trombina, Protrombina, Trombose, Neoplasias colorretais

## Abstract

**BACKGROUND:**

The relationship between thrombosis and cancer is based on evidence that cancer promotes prothrombotic changes in the host hemostatic system. The activation of blood coagulation is closely linked to tumor growth and dissemination.

**AIMS:**

To evaluate whether quantifications of plasma circulation tumor deoxyribonucleic acid (DNA) and thrombin-antithrombin complex could act as predictors for thrombotic events and death in patients with gastric or colorectal adenocarcinomas, while also evaluating the Karnofsky Performance Status.

**METHODS:**

Eighty-two patients were included in the study and divided into three groups: controls (n=20), gastric adenocarcinomas (n=21), and colorectal adenocarcinomas (n=41). In order to calculate the Karnofsky index, information was collected to measure the patient’s ability to perform common daily tasks. The following serum measurements were conducted: complete blood count, platelet count, extracellular deoxyribonucleic acid, and thrombin-antithrombin complex.

**RESULTS:**

Ten patients (16%) experienced thrombosis during treatment. Patients with thrombin-antithrombin complex levels greater than 0.53 had a five-times higher risk of thrombosis. Lower Karnofsky Performance Status was also a risk factor for the event in this population. Neither thrombin-antithrombin complex nor plasma circulation tumor DNA were predictors of death after multivariate adjustment. Thus, Karnofsky index signaled a better overall survival prognosis for colorectal and gastric adenocarcinoma patients.

**CONCLUSIONS:**

Thrombin-antithrombin complex acts as a marker for thrombosis in patients with colorectal and gastric adenocarcinomas. We recommend prophylactic anticoagulation when the Karnofsky value is low and/or the thrombin-antithrombin complex concentration is greater than 0.53 ng/ml.

## RESUMO

**RACIONAL:**

A relação entre trombose e câncer é baseada em evidências de que o câncer promove alterações protrombóticas no sistema hemostático do hospedeiro. A ativação da coagulação sanguínea está intimamente ligada ao crescimento e disseminação do tumor.

**OBJETIVOS:**

Avaliar se as quantificações do ácido desoxirribonucleico (DNA) tumoral na circulação plasmática e do complexo trombina-antitrombina poderiam atuar como preditores de eventos trombóticos e morte em pacientes com adenocarcinomas gástricos ou colorretais, avaliando também o status de desempenho de Karnofsky.

**MÉTODOS:**

Oitenta e dois pacientes foram incluídos no estudo e divididos em três grupos: controles (n=20), adenocarcinomas gástricos (n=21) e adenocarcinomas colorretais (n=41). No cálculo do índice de Karnofsky foram coletadas informações para medir a capacidade do paciente de realizar tarefas diárias comuns. As seguintes dosagens séricas foram realizadas: hemograma completo, contagem de plaquetas, ácido desoxirribonucleico extracelular e complexo trombina-antitrombina.

**RESULTADOS:**

Dez pacientes (16%) apresentaram trombose durante o tratamento. Pacientes com níveis de complexo trombina-antitrombina superiores a 0,53 apresentaram risco cinco vezes maior de trombose. A baixa classificação de desempenho de Karnofsky também foi considerada um fator de risco para o evento nesta população. Nem o complexo trombina-antitrombina, nem o DNA tumoral da circulação plasmática foram preditores de morte após ajuste multivariado. O índice Karnofsky sinalizou um melhor prognóstico de sobrevida global para pacientes com adenocarcinoma colorretal e gástrico.

**CONCLUSÕES:**

O complexo trombina-antitrombina atua como marcador de trombose em pacientes com adenocarcinoma colorretal e gástrico. Recomendamos anticoagulação profilática quando o valor do Karnofsky for baixo e/ou a concentração do complexo trombina-antitrombina for superior a 0,53 ng/ml.

## INTRODUCTION

Thrombosis is currently recognized as a major public health problem, as it is responsible for 10% of hospital deaths and is the second leading cause of death among cancer patients^3^. In some countries, the incidence of deaths from thrombosis is higher than the combined incidence of breast cancer, acquired immunodeficiency syndrome (AIDS), and car accidents^2^. Thrombosis not only causes mortality but also morbidity and results in the patient’s absence from normal activities, leading to a significant decrease in quality of life^4^.

The relationship between thrombosis and cancer is based on evidence that cancer promotes prothrombotic changes in the host hemostatic system. The activation of blood coagulation is closely linked to tumor growth and dissemination^5,6^. The main mechanisms of cancer-related thrombosis include the expression of procoagulant factors on tumor cells, the release of inflammatory cytokines, and pro-angiogenic factors, such as vascular endothelial growth factor. These same properties also correlate with cancer progression^7,8^.

The coagulation system interacts with immune and inflammatory responses, tissue healing, and angiogenesis^1^. Cancer patients with more aggressive tumor behavior are at higher risk for thrombosis^9^. The incidence rate in the general population is one to two cases per 1,000 persons/year, while patients with malignant diseases usually have a four to ten times higher risk, which may increase even more, particularly in patients with brain and pancreatic cancer^10-12^.

Current evidence suggests that the absolute risk for venous thromboembolism (VTE) depends on tumor type, cancer stage, and treatment with antineoplastic agents. VTE includes a spectrum of clinical pictures ranging from superficial and deep venous thrombosis (DVT) to pulmonary embolism (PE)^13,14,28^. One in every seven patients with cancer dies due to complications, especially during hospital stay. Of these, 60% have single-site cancer or limited metastatic disease. According to Guedes et al., patients may survive longer when they do not have DVT or PE^15^.

Rates of VTE may differ even among diverse histologic types of disease involving the same organ, with adenocarcinomas typically being associated with a higher risk of VTE than squamous cell carcinomas and being more frequent in the presence of malignant gastrointestinal neoplastic disease^16^.

It is now known that thrombosis has a multifactorial origin, and several other risk factors (both hereditary and acquired) may be present. However, identifying hereditary thrombophilia is not required for a thrombotic event to occur^17^. Many patients only present risk factors acquired during the thrombotic event. Coagulopathies are linked to the inflammatory, endocrine, and immune systems, and any change in this balance predisposes to alterations^18^.

The thrombin-antithrombin complex (TAT) and the expression of plasma circulating tumor DNA (ctDNA) have the potential to detect thromboembolic risk for the patient^1,23^. Thus, both markers of thrombosis may be useful in aiding the prevention of complications of this disease and contributing to a better assessment of the prognosis of patients affected by the digestive system cancer^26,39^.

This prospective observational study aimed to evaluate whether quantifications of plasma ctDNA and TAT could act as predictors of thrombotic events and death in patients with gastric or colorectal adenocarcinomas, while also evaluating the Karnofsky Performance Status (KPS).

## METHODS

The project was approved by the Research Ethics Committee of the National Cancer Institute (INCA/RJ, *Instituto Nacional do Câncer do Rio de Janeiro*), under nº CAAE 15367013.2.0000.5274, technical advice nº 4.231.089. All study patients signed the Informed Consent Form. An epidemiological questionnaire was applied in which the presence of risk factors for thrombosis was verified.

Sixty-two patients with colorectal and gastric adenocarcinoma, confirmed by histopathological diagnosis, were included in the study between August 2013 and June 2014 at INCA. Outpatient follow-up and medical records were maintained for six years or until death, or until the date of last consultation, as long as they occurred within six years from the insertion into the study.

### Criteria for inclusion in the study

1. Not having undergone antineoplastic treatment;
2. Having active neoplastic disease;
3. Being of age equal to or greater than 18 years;
4. Having malignant neoplasm of the stomach or colorectal.

### Exclusion criteria

1. Patients with poor understanding or social inability to follow the study;
2. Being on anticoagulants or platelet aggregation inhibitors.

### Clinical evaluation

The following morbidities were considered:

1. Systemic arterial hypertension and diabetes mellitus;
2. Oral contraceptive and hormone replacement therapy;
3. Previous medical history of deep vein thrombosis and family history.

### Laboratory assessment

The following exams were collected and analyzed:

1. Complete blood count;
2. Thrombin-antithrombin complex (TAT);
3. Plasma circulating tumor DNA (ctDNA).

### Assessment of risks and benefits

The only risks related to the study were those due to venipuncture, such as local redness, bruising, and some discomfort, which are usually transient.

The benefit of the study for the patients is that they will be better monitored for the appearance of thrombosis (with Doppler), which may help to diagnose and treat thrombosis with a few symptoms and reduce the risk of pulmonary embolism. It may also help to understand the mechanism of thrombosis in patients with these types of cancer, providing useful information for thrombosis prevention protocols and for the use of markers with good sensitivity and specificity to assess the risk of thrombosis.

A blood sample was collected from all patients (two tubes with citrate, one tube with ethylenediaminetetraacetic acid (EDTA), and one tube without anticoagulant) in order to measure the complete blood count, platelet count, trypsinogen activation peptide (TAP) (ELISA kit, Enzyme Research - USA), and partial thromboplastin time (PTT) (Cell Dyn Ruby, Abbott and XE-2100, Roche), using the manufacturer’s recommendations.

The quantification of circulating DNA was performed on platelet-poor plasma obtained from selected cancer patients and healthy donors. The analysis was conducted using flow cytometry and followed the protocol described by Butt and Swaminathan^5^. Circulating DNA quantification was analyzed for non-fluorimetric parameters of size and granularity measured in ng/ml (forward scatter – FSC x side scatter – SSC) using a FACScan BD (Becton & Dickinson) with a total of 10,000 events per sample. The ExoQuick™ reagent isolation method (System Biosciences) was used^5,37^.

### Epidemiological questionnaire

An epidemiological questionnaire was administered at the time of diagnosis, prior to treatment commencing. This questionnaire verified the presence of risk factors for thrombosis such as previous thrombosis, stroke and infarction before age 55, previous abortions, past medical history of thrombosis, family history of thrombosis, and use of medication (e.g., hormones).

### Karnofsky performance scale index

The KPS index was utilized in this dissertation. It is a rating scale that assesses the impact of the disease on daily activities, self-care, work, and the need for care. It ranges from 0 (imminent death) to 100 (no complaints or signs of illness) and represents normal functioning (100) to death (0) in ten-point increments (Table 1).

**Table 1:** Karnofsky performance scale^38^.

Classification (%)	
100	No signs or complaints, no evidence of illness.
90	Minimal signs and symptoms, able to perform activities with effort.
80	Major signs and symptoms, performs activities with effort.
70	Takes care of himself, not able to work.
60	Needs occasional assistance, able to work.
50	Requires considerable assistance and frequent medical care.
40	Needs special medical care.
30	Extremely incapacitated, needs hospitalization, but with no imminent death.
20	Very sick, needs support.
10	Dying, imminent death.

### TNM staging

The TNM staging system, developed by the American Joint Committee on Cancer, was employed to describe the location, size, and spread of cancer, as well as its effect on other organs.

### Statistical analysis

Categorical data (proportions) were analyzed using Pearson’s chi-square test. The Tamhane T2 test was applied to compare interval variables, while the Student’s *t-*test was used for continuous variables with a normal distribution. Non-parametric Mann-Whitney test was conducted in cases where data distribution was not normal. The Smirnov-Kolmogorov test was used to test data normality. A p<0.05 was considered significant.

The Kaplan-Meier curve was adopted to estimate the overall survival probability of patients with or without metastases. Follow-up time was calculated from the date of enrollment in the study until the end of the inclusion period, which was six years after the study’s inception. Deaths during this period were recorded, as well as outpatient follow-up losses. The Cox proportional hazards model was used to calculate the death hazard ratio.

Logistic regression models (univariate and multivariate) were employed to assess the risk factors for the development of thrombosis. The criteria for inclusion of independent variables in the multivariate regression models were based on the results of the univariate analysis and clinical relevance. Independent variables included in the models were: sex, race, histological type, KPS, staging, smoking, hypertension, family history, past medical history, use of oral contraceptives, hemoglobin, leukocytes, and platelets. Variables with a p<0.20 were selected as candidates for multivariate analysis after identifying possible collinearities between them through correlation analysis. The backward elimination method was used to obtain a parsimonious predictive model.

Data were entered into an Excel^®^ spreadsheet and subsequently exported to the Statistical Package for Social Sciences (SPSS, version 13.0) program.

## RESULTS

### Socio-demographic characteristics and history of previous diseases

Table 2 presents the socio-demographic characteristics of our sample. In the present study, a total of 62 individuals were analyzed and grouped into colorectal adenocarcinoma (n=42) and gastric adenocarcinoma (n=20).

**Table 2:** Socio-demographic characteristics and history of previous diseases.

Variables	n	%
Gender		
Male	30	48.52
Female	32	51.47
Adenocarcinoma		
Stomach	20	32.30
Rectal colon	42	67.70
Metastasis (n=57)		
Yes	18	31.60
No	39	68.40
Missing data	5	
Thrombosis		
Yes	12	19.40
No	50	80.60
Staging (n=57)		
Staging 1	3	5.20
Staging 2	10	17.54
Staging 3	20	35.08
Staging 4	24	42.10
Missing data	5	
Color (n=60)		
White	31	51.70
Non-white	29	48.30
Missing data (n=2) COPD		
Yes	7	11.30
No	55	89.70
SMO		
Yes	34	54.80
No	28	45.20
SAH		
Yes	39	62.90
No	23	30.10
DM		
Yes	8	12.90
No	54	87.10
FH (n=60)		
Yes	27	45.00
No	33	55.00
Missing data	2	
PMH		
Yes	20	32.30
No	42	67.70
OC/Hormone		
Yes	5	8.10
No	57	91.90

COPD: chronic obstructive pulmonary disease; SMO: smoker, DM: diabetes mellitus; SAH: systemic arterial hypertension; FH: family history; PMH: past medical history; OC: oral contraceptive.

A similar distribution regarding tumors was observed between men (n=30) and women (n=32). Of the 57 patients (information was not available for five of the original 62), 18 had metastases (31.6%). Regarding tumor size, we found T1 in three patients, T2 in ten patients, T3 in 20 patients, and T4 in 24 patients (information was not available for five). Of the total 62 patients, 12 had thrombosis at some time during the study.

Thirty-one patients were white and 29 were brown or black (information not available for two). Among patients with colorectal adenocarcinoma, 19 (45.2%) were self-reported as white, and 22 (52.4%) as non-white. For those with gastric adenocarcinoma, 11 (55%) self-reported as white and ten (45%) as non-white.

In the sample, seven patients had chronic obstructive pulmonary disease (COPD) and 34 were smokers. Of the 62 patients, 39 had systemic arterial hypertension and eight had type II diabetes mellitus.

Family history of thrombotic events (DVT, miscarriage, infarction and/or stroke in co-blood siblings under 55 years of age) was positive in 27 of the 60 patients who could answer the epidemiological questionnaire.

In the past medical history, 20 patients had been diagnosed with prior thrombosis at some point in their lives and were treated. However, they were not treated with heparin or platelet antiaggregants at the time of study initiation. Five of these patients reported using oral contraceptives or hormone replacement such as levothyroxine.

### Description of the continuous variables

Table 3 describes the continuous variables of the 62 study patients. The median TAT was 0.46 ng/ml ranging from 0.02–18.04 in patients. The median DNA was 237.04 ng/ml ranging from 155–715. Median hemoglobin was 12.00 g/dL, the median white blood cell count was 8,275 μL, and the median platelet count was 284,500 μL.

**Table 3:** Variable data index.

Variable	Median (Min-Max)	IQR (Q3-Q1)
KPS (n=58)	80 (20–100)	20
TAT	0.46 (0.02–18.04)	1.20
DNA (n=61)	237.04 (155–715)	189
Hemoglobin (n=61)	12.00 (5.21–15.60)	3.25
Leukocytes	8,275 (4,040–24,680)	2,995
Platelets	284,500 (33,000–921,000)	161,000

IQR: interquartile ratio; KPS: Karnofsky performance status; TAT: thrombin-antithrombin; DNA: deoxyribonucleic acid.

### Thrombin-antithrombin complex and deoxyribonucleic acid measurements among patients with colorectal adenocarcinoma, stomach adenocarcinoma, and controls

**Figure 1:**
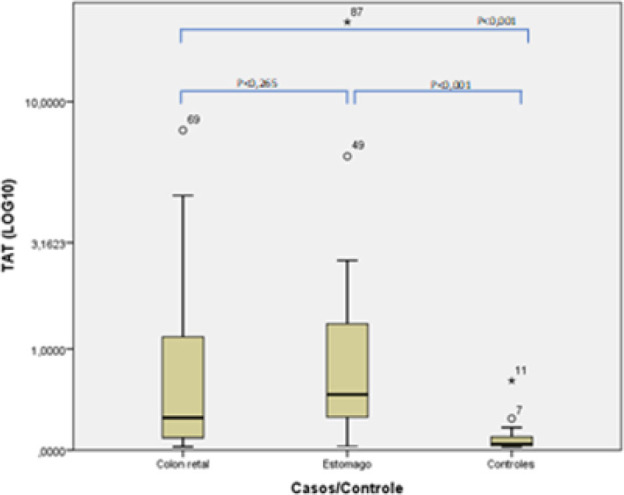
Measurement of the thrombin-antithrombin complex in the control, colorectal adenocarcinoma, and gastric adenocarcinoma groups.

As seen in Figure 1, a comparison of the median TAT scores between the colorectal adenocarcinoma group and the gastric adenocarcinoma group showed no significant difference. However, a p<0.001 was obtained when the results of both groups were compared to those of the control group.

**Figure 2:**
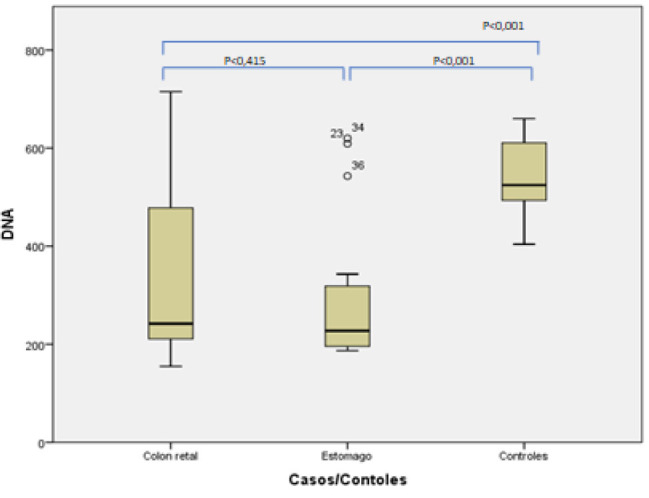
DNA measurement in the colorectal adenocarcinoma, gastric adenocarcinoma, and control groups.

A comparison of the median DNA levels between the groups of colorectal adenocarcinoma and gastric adenocarcinoma showed no significant difference. However, once again, when the results of both groups were compared to those of the control, a significant increase (p<0.001) was observed (Figure 2).

Differences were observed between the groups with cancer and the control. The horizontal bars represent the medians, the boxes show the 25th and 75th percentiles, and the vertical bars indicate the variation, comprising the means and standard deviation of each group. The significance level was set at p<0.05.

### Survival analysis for cancer patients

The probability of overall survival at six years between patients with and without metastases, assessed by Kaplan-Meier, was significantly lower in the groups with metastatic tumors at diagnosis (p<0.001).

We also observed a lower overall survival probability in patients with gastric adenocarcinoma than in those with colorectal adenocarcinoma (p=0.02).

There was a lower probability of overall survival at six years in adenocarcinoma patients with DNA=400 ng/μl (53.3%) compared to DNA=400 ng/μl (28.3%), as evidenced by p=0.05.

### Analysis of prognostic factors for risk of death

Data on socio-demographic characteristics and history of past illnesses from the KPS were used to assess the risk of death (Table 4).

**Table 4:** Prognostic factors for risk of death.

Variables	Nº	Univariate analysis		Multivariate analysis	
		HR	p	HR	p
TAT	62	1.053 (0.958–1.158)	0.281	0.957 (0.854–1.073)	0.455
DNA	61	0.999 (0.996–1.001)	0.172		
DNA <400					
No	15	1			
Yes	46	2.182 (0.963–4.944)	0.061	1.415 (0.547–3.659)	0.473
Gender					
Male	30	1			
Female	32	1.244 (0.673–2.300)	0.463		
Tumor type					
Colon-rectum cancer	42	1		1	
Stomach cancer	20	2.612 (1.380–4.943)	0.003	2.866 (1.386–5.923)	0.004
Metastasis (n=57)					
No	39	1		1	
Yes	18	2.812 (1.448–5.463)	0.002	3.082 (1.461–6.501)	0.003
Thrombosis					
No	50	1			
Yes	12	1.693 (0.827–3.466)	0.150		
Tumor staging (n=57)					
T1 (ref)	3	1			
T2	10	1.255 (0.260–6.058)	0.777		
T3	20	0.805 (0.180–3.606)	0.777		
T4	24	0.924 (0.212–4.025)	0.916		
KPS	58	0.968 (0.945–0.991)	0.006	0.972 (0.946–0.998)	0.033
KPS (n=50)	58				
No	55				
Yes	3	12.575 (3.260–48.506)	<0.001		
Color (n=60)					
White	31	1			
Non-white	29	0.957 (0.514–1.780)	0.889		
COPD					
No	55	1			
Yes	7	0.989 (0.387–2.524)	0.981		
SMO		1.065 (0.576–1.968)	0.842		
No	28	1			
Yes	34	1.060 (0.574–1.959)	0.852		
SAH					
No	23	1			
Yes	39	0.824 (0.439–1.548)	0.548		
DM					
No	54	1			
Yes	8	1.661 (0.697–3.954)	0.252		
FH (n=60)					
No	33	1			
Yes	27	1.097 (0.593–2.026)	0.769		
PPH					
No	42	1			
Yes	20	0.720 (0.367–1.413)	0.339		
OC/Hormone					
No	57	1			
Yes	5	1.369 (0.488–3.845)	0.551		
HB	61	0.928 (0.828–1.040)	0.198*		
Leukocyte count	62	1.000 (1.000–1.000)	0.332		
Platelet count (>580,000)	62	1.000 (1.000–1.000)	0.057		
No	52			1	
Yes	4	3.231 (1.142–9.147)	0.027	3.533 (1.137–10.978)	0.029

HR: hazard ratio; p: p-value; COPD: chronic obstructive pulmonary disease; SMO: smoker; SAH: hypertension; DM: diabetes mellitus; FH: family history; PPH: previous pathological history; OC: oral contraceptive; HB: hemoglobin; Plat: platelets; KPS: Karnofsky Performance Status; TAT: thrombin-antithrombin complex; DNA: deoxyribonucleic acid; T: tumor.

In multivariate analysis, TAT and DNA variables were not statistically significant in predicting the risk of death when controlled for factors measured at diagnosis: tumor type, presence of metastasis, KPS (analyzed as a continuous variable), and platelets >580,000.

In the final model, gastric tumor (p=0.004), platelets greater than 580,000 (p=0.057), presence of metastasis at diagnosis (p=0.003), and low KPS were significant outcomes related to the risk of death.

### Analysis of risk factors for thrombosis in tumors

Table 5 (as mentioned in the Discussion) shows the multivariate analysis of the risk of developing thrombosis. The dichotomized TAT variable with a value >0.53 revealed a five-fold increased risk of developing thrombosis; odds ratio (OR)=5.018 (1.004–25.091) when analyzed along with the parameters KPS, FH, and COPD. In the final model, the variable TAT >0.53 ng/mL was associated with an increased risk of developing thrombosis. The higher the KPS, the lower the risk of developing thrombosis, OR=0.974 (0.958–0.990), p=0.002. Positive family history of thrombosis and patients with COPD as comorbidities were also associated with an increased risk of thrombosis (Table 5).

**Table 5:** Risk factors for thrombosis.

Variables	Nº	Univariate analysis		Multivariate analysis	
		OR	p	OR	p
TAT	62	1.179 (0.940–1.478)	0.154		
DNA	61	1.000 (0.996–1.004)	0.985		
TAT >0.53					
No		1		1	
Yes		3.000 (0.796–11.308)	0.105	5.018 (1.004–25.091)	0.049
Gender					
Male	30	1			
Female	32	0.714 (0.200–2.555)	0.605		
Tumor type					
Colon-rectum cancer	42	1			
Stomach cancer	20	0.941 (0.247–3.592)	0.929		
Metastasis (n=57)					
No	39	1			
Yes	18	0.417 (0.80–2.165)	0.298		
Tumor staging (n=57)					
T1/T2	13	1			
T3/T4	44	0.741 (0.165–3.321)	0.695		
KPS	62	0.966 (0.926–1.008)	0.108	0.974 (0.958–0.990)	0.002
Color (n=60)					
White	31	1			
Non-white	29	0.460 (0.122-1.734)	0.251		
COPD					
No	55	1		1	
Yes	7	3.833(0.730–20.1340	0.112	5.920 (0.769–45.553)	0.088
SMO					
No	28	1			
Yes	34	1.846 (0.492–6927)	0.363		
SAH					
No	23	1			
Yes	39	2.000 (0.482– 8.306)	0.340		
FH (n=60)					
No	33	1		1	
Yes	27	0.333 (0.080–1.384)	0.130	0.160 (0.025–1.010)	0.051
PMH					
No	42	2			
Yes	20	0.600 (0.164–2.196)	0.440		
OC/Hormone					
No	57				
Yes	5				
HB	61	0.980 (0.770–1.247)	0.869		
Leukocyte count	62	1.000 (1.000–1.000)	0.452		
Platelet count	62	1.000 (1.000–1.000)	0.374		

COPD: chronic obstructive pulmonary disease; SMO: smoker; SAH: hypertension; DM: diabetes mellitus; HF: family history; PMH: previous medical history; OC: oral contraceptives; HB: hemoglobin; Plaq: platelets; KPS: Karnofsky performance status; TAT: thrombin-antithrombin complex; DNA: deoxyribonucleic acid; T: tumor.

The correlation between the KPS index and TAT concentrations >0.53 ng/mL demonstrated that a lower KPS was associated with a higher probability of thrombosis. A progressive increase in the probability of thrombosis was observed, with an inverse relationship to the KPS index.

## DISCUSSION

Colorectal cancer is the third most commonly diagnosed cancer worldwide, with over 1.9 million new cases annually^41^. Rectal cancer is the eighth most common cancer according to the Global Cancer Observatory (GLOBOCAN) 2018. Colorectal cancer is more frequently diagnosed among males than females in countries around the world, with no country reporting higher rates of colorectal cancer among females^38^. In Brazil, gastric cancer, with adenocarcinoma accounting for 95% of cases, is the third most frequent cancer among men and the fifth among women^32,42^.

For solid tumors, biopsy remains the gold standard for pathological and immunohistochemical diagnosis, which can impact patient prognosis. However, this method is invasive, expensive, and carries a risk of complications such as tumor dissemination, vessel perforation, bleeding, infection, and thrombosis, among others^40^. Additionally, it is possible to obtain an inadequate quantity of material^31^.

The connection between cell-free DNA (cfDNA) and cancer has been established for more than four decades, with increased concentrations of cfDNA detected in various types of cancer that are proportional to staging^42^.

Liquid biopsy is a safer, more economical, and non-invasive method for evaluating tumor cells and has been incorporated as the best alternative in recent years. In liquid biopsy, peripheral blood or other body fluids are collected from the patient, from which various cells and components can be isolated, including cfDNA released from cells by active secretion and apoptosis^20^. Healthy individuals have between 1 and 10 nanograms of cfDNA in each milliliter of blood, with the main components originating from hematopoietic cells^22^ (Table 6).

**Table 6:** A comparison of applications between solid tissue biopsy and cell-free DNA31.

Features	Biopsy	cfDNA
Able to observe histological patterns	Yes	No
Easy to obtain with minimal discomfort	No	Yes
Capacity to represent the complete mutation spectrum of the tumor	No	Yes
Evaluation of RNA expression.	Yes	No
Detection of SNVs, CNVs, indels, SVs	Yes	Yes
Proteomic analysis	Yes	No
Analysis of methylation patterns	Yes	Yes
Potential to detect MRD	No	Yes

SNV: single nucleotide variant; CNV: code number variant; indel: insertion/exclusion; SV: structural variant; MRD: minimal residual disease; RNA: ribonucleic acid; cfDNA: cell-free DNA.

Some authors only describe a correlation between the amount of cfDNA and cancer staging (higher staging and presence of metastasis correspond to higher DNA levels), rather than with the type of primary tumor. Moreover, changes in cfDNA levels can indicate the response to treatment. High or increasing concentrations of cfDNA can signify tumor enlargement and predict disease relapse. Thus, cfDNA presents a favorable alternative for tumor follow-up and therapy change if necessary.

The present study showed no significant difference in DNA medians between colorectal and gastric adenocarcinomas. Still, there was a significant increase (p<0.001) when the results were compared to those of the control group. Furthermore, it was observed that serum measurements with results ≥400 ng/μl correlated with a higher risk of death, almost double that found in patients with concentrations of 400 ng/μl (53.3 vs. 28.3%, p=0.05).

Volik et al. reported that cfDNA levels can express both tumor burden and biological mechanisms, providing a multifaceted picture of the disease^37^. The usefulness of a prognostic parameter has been attested and can facilitate the follow-up of disease progression and even assist in the evaluation of the patient’s response to treatment.

In the absence of vascularization, primary tumors and metastases do not grow beyond 2 to 3 mm^3^ in size. Therefore, angiogenesis is fundamental for tumor growth, and the pro-coagulant activity of the Tissue Factor stimulates the production of thrombin, which promotes tumor angiogenesis^21^. In this study, 19.35% of oncology patients developed thrombosis at some time during treatment, consistent with the literature which reports a rate of around 20%.

One way to estimate thrombin production is to measure the final product, TAT. The concentration of TAT III was about ten times higher in patients with adenocarcinomas (colorectal 0.47 ng/mL and gastric 0.46 ng/mL) compared to those of the control group (0.045 ng/mL), with a significant difference of p<0.001. Khorana has described that tumors located in the stomach increase the patient’s risk and, in his study, gastric adenocarcinoma increased the risk of death in patients (p=0.004, p<0.05).

The author also related the increase in TAT as a predictive factor for VTE in cancer patients. TAT also showed a predictive value for thrombosis in the sample studied when results >0.53 ng/mL were obtained, with a five-fold increased risk of patients developing this complication. Based on these data, it is suggested that prophylactic anticoagulation should be instituted when TAT concentrations reach this level^29^.

In the present study, it was observed that in a stomach tumor, a platelet count >580,000/μL increases the risk of death. Khorana reported that an elevation in the pre-chemotherapy platelet count would increase the risk of VTE^34^. There is already evidence that patients with DVT and cancer have shorter survival than those with cancer without thrombosis^27^.

This is a sign of poor prognosis that burdens treatment and is associated with high morbidity and mortality, reduced quality of life, and may lead to treatment discontinuation^35^. It was found that thromboprophylaxis reduces VTE risk; however, oncologists usually do not indicate outpatient prophylaxis for fear of bleeding and because the subcutaneous application is uncomfortable^33^.

As mentioned earlier, a Medicare database study of more than eight million patients without cancer and one million patients with cancer showed that cancer patients diagnosed with associated thrombosis had a much higher risk of dying from cancer in the subsequent six months of follow-up than cancer patients without thrombosis^24^.

The importance of this study lies in the possible complications that emerge during hospitalization, which can worsen the patient’s clinical picture by reducing mobility, increasing insulin resistance, anxiety, depression, cardiovascular deconditioning, DVT, and/or pulmonary thromboembolism. The risk of thromboembolism, the leading cause of death in cancer patients, increases if the cancer patient remains bed-bound for more than three days^40^.

In the present sample, among the patients analyzed following the TNM classification, 31.57% had metastases. According to the literature, TNM classification is essential for defining oncological treatment and percentage of survival besides being related to thromboprophylaxis for hospitalized patients.

The KPS evaluation showed that the higher the percentage obtained (that is, the less impairment to the performance of daily activities), the greater the survival rate. When patients need only occasional assistance and can work (KPS>60%), the prognosis is better. Multivariate analysis has also shown, unprecedented in the literature on the subject, that KPS <60% increases the risk of thrombosis. Thus, prophylactic anticoagulation would be indicated in these cases^19^.

Family history of thrombotic events (DVT, abortion, infarction, and/or stroke in co-sanguineous siblings under 55 years of age) was present in 45% of the patients analyzed. Patients who have first-degree relatives with a history of VTE are twice as likely to acquire VTE. The chances increase to four times if there is more than one first-degree relative with a positive family history^36^.

Other risk factors for thrombosis and death of patients with gastrointestinal cancer were analyzed in this paper with the aim of evaluating possible confounding variables to adjust the effect of TAT and ctDNA^33^. Although our patients had a very low incidence of thrombosis, they showed increased thrombin production compared to the group without cancer. These findings suggest that an important factor in preventing thrombosis may be the activation of fibrinolysis. If these findings are confirmed, they could have an essential impact on patient treatment, since anti-fibrinolysis drugs could induce thrombosis. According to current studies, this thrombin may also promote cancer growth by interfering with angiogenesis and activating protease-activating receptors^25^.

This highlights the importance of considering prophylaxis for patients in high-risk situations to prevent and reduce morbidity and mortality, as well as identifying pro-thrombotic alterations to avoid coagulation disorders^31^.

In this study, we emphasized the importance of KPS, quantification of TAT, and ctDNA correlation to better prognosis, longer survival, and state of hypercoagulability, while considering staging and incidence of death for patients with colorectal and gastric neoplasms^30^.

## CONCLUSION

A higher KPS signals a better prognosis for patients with colorectal and gastric adenocarcinoma. DNA expression plays a role in these patients, with measurements ≥400 ng/μl correlating with an increased risk of death. TAT acts as a marker for thrombosis in colorectal and gastric adenocarcinoma patients, with the risk increasing when measurements are >0.53 ng/ml. Based on the obtained data, prophylactic anticoagulation is recommended when KPS <60% and TAT >0.53 ng/ml.

The evidence supports the recommendation for the use of prophylactic anticoagulation when TAT >0.53 ng/ml and KPS is lower. Further studies with a larger number of patients and greater variety of tumors are needed to confirm the findings on the prognostic value regarding survival and the risk of thrombosis of TAT and cfDNA.
